# Analysis of solutions for a blockchain compliance with GDPR

**DOI:** 10.1038/s41598-022-19341-y

**Published:** 2022-09-02

**Authors:** Mateusz Godyn, Michal Kedziora, Yingying Ren, Yongxin Liu, Houbing Herbert Song

**Affiliations:** 1 Department of Applied Informatics, Wroclaw University of Science and Technology, Wroclaw, Poland; 2 Security and Optimization for Networked Globe Laboratory (SONG Lab), Embry-Riddle Aeronautical University, Daytona Beach, 32114 USA

**Keywords:** Computer science, Information technology

## Abstract

The aim of this paper was to perform an analysis of the state-of-the-art solutions of the permissioned blockchain compliance with the General Data Protection Regulation (GDPR), including the implementation of one of the analyzed methods and the own solution. This paper covers the subject of GDPR and its impact on already existing blockchain databases to determine the domain of the problem, including the necessity to introduce mutability in the data structure to comply with the ”right to be forgotten”. The performed analysis made it possible to discuss current research in technical terms as well as in the regulation itself. In the experimental part, attempts were made to research and implement the Reference-based Tree Structure (RBTS), including the performance tests. The proposed solution is efficient and easily reproducible. The deletion of unwanted content is quick and requires consent only from the owner of personal data; therefore, eliminating the dependency on the other blockchain network participants.

## Introduction

Until recently, the core advantage of blockchain was immutability. Now, with the recent General Data Protection Regulation (GDPR), it has also started to be its weakness. The ’Right to be Forgotten’, which is part of GDPR, enforces the possibility of deleting or anonymizing personal data from the blockchain, which is against the design of the technology and its immutability principle. Not complying with the law can lead to serious financial penalties, so many companies and organizations fall back from plans to incorporate blockchain into their system^1,2^. On the other hand, the solution must still meet both security and efficiency requirements^3,4^. There is no verified and flexible solution to this kind of problem, so the growth of blockchain has slowed^5^.

The aim of the paper is to analyze already proposed methods for achieving GDPR-compliant blockchain in a permissioned setting and then implement proof-of-concept for one of the analyzed methods, and experiment on it with a newly proposed approach.

The remainder of this paper is structured as follows. First, the General Data Protection Regulation is briefly introduced with an explanation of its importance and the way it impacts blockchain systems. Afterwards, the related work to the GDPR-compliance problem is discussed, followed by attempts to recreate it. In the end, a proposed Reference-based Tree Structure (RBTS) is presented with its theoretical background, implementation, and tests, achieving high reproducibility and excellent results of experiments on it, which enable researchers to determine areas for future development of the solution. The results of the implementation are shown and covered. The article is concluded by describing the research reproducibility aspect of the work. Lastly, areas for future improvements for the proposed solution are discussed.

### Analysis of blockchain compliance with GDPR principles

With advancement in technology, data flow between systems of all kinds is essential. These data may consist of sensitive records, such as personal data, which should be treated with special care. Permissionless access to this kind of data results in a violation of the right to privacy that is part of the European Convention on Human Rights^6^. Due to the convention’s age, it was required to make a regulation taking into account computerization. That is how *General Data Protection Regulation* has formed on April 27, 2016, which was eventually put into effect on May 25, 2018^7^. In general, it can be said that data should be processed explicitly, collected only for specified purposes^8^. The dataset should contain only necessary data and shall be stored as long as it is needed for a specified purpose. It is worth noting that the stored records have to be secured in order to prevent unauthorized processing, accidental loss, destruction, or damage (integrity and confidentiality principle)^9^.

People who share their data on the blockchain can have access to the shared data and check how it is processed without obstacles when it comes to public blockchains. It is also possible to design such permissioned ones that can fulfill the need for this transparency^10^ by sharing how much data is necessary for blockchain and what its purpose is for which the creator of the distributed database can answer themselves. Again, this is more applicable to more restricted systems, whereas the more open ones can prove their point through an open-source code or access to the insights of various processes, for example, of sent transactions between users. Regarding the storage limitation rule, one can state that the data is needed as long as the blockchain exists, as, without it, it cannot function properly^11^.

The first problems that may arise are bound to the confidentiality rule^12^. Unauthorized or unlawful processing of personal data can be made impossible on restricted and permissioned blockchains. However, public blockchains must take some steps to secure data with appropriate measures, such as using encryption or anonymization^13^. It is still questionable whether the data in blockchain can fall under the term of the ’household exemption’ of GDPR (Article 2). This would allow systems to store specific personal data without special security treatment^14^. The Commission Nationale de l’Informatique et des Libertés (CNIL), the French Data Protection Authority, as a matter of fact, announced guidance on blockchains, where it showed the application of the household exemption on the example of Bitcoin. However, the context of blockchain can be professional or commercial and therefore fall short in light of Article 2 of GDPR, the permissionless blockchain can be used for private purposes, and finally, not all data are excluded from GDPR even when the household exemption rule applies^15^.

The accountability principle of GDPR is even more burdensome, as this rule assumes existence of controller and many blockchains seek to achieve decentralization. Determining who can fall under the umbrella of the term ’controller’ seems impossible^16^.

Even if one could create a blockchain compliant with presented GDPR’s principles and defend their work, ultimately it would not be possible to reconcile the blockchain with Article 17 of GDPR—the right to erasure, often referred to as the *right to be forgotten*^17^. In summary, a person whose data is being processed has the right to ’obtain from the controller the erasure of personal data concerning him or her without undue delay’^7^. This right can be used in the form of sudden revocation of the agreement to process the data. In this situation, the controller has to take the necessary steps in order to delete the person’s data. As previously discussed, it might be impossible to find a controller and even if the one is found, they will not be able to destroy the data without deleting the whole blockchain. To make the blockchain comply with GDPR and not be susceptible to deletion, there should be a way to manipulate the data in it, which is a difficult problem because the core of the blockchain is immutability: without it, chains of blocks cannot be validated and therefore stored as true in a distributed network^18^.

At first glance, one might think that making blockchain fully compatible with the new regulation is unachievable, and any attempts to do so are meaningless, as immutability is a core of this kind of distributed database. Fortunately, it turns out that there is research that attempts to make this happen and proposes solutions. Not all of them are efficient, but they reveal a possible path worth considering for other future proposals to resolving the problem. The common goal in most approaches is to enable some kind of data deletion from the blockchain or its alteration.

### Related work

Enabling blockchain compliance with GDPR is a subject of growing importance. In real-life applications, it is an even more complex topic and it can be presented from the perspective of cybersecurity^19,20^, healthcare^21,22^, Industrial IoT^23,24^ or public services^25^. So far only a handful of ideas have been presented that endue some kinds of modification to the blockchain structure in the form of alteration or deletion blocks or transactions. In this section, the most promising ideas are briefly covered by pointing out their strengths and weaknesses.

#### Chameleon hash

Back in 2017, Ateniese et al.^26^ had focused on allowing modification of data contained in blocks and retaining the structure of current blockchain designs. Their concept consists of the usage of a more secure chameleon hash previously described by Ateniese and Breno^27^ in 2004 and the usage of asymmetric cryptography.

The chameleon hash is a pretty popular tool when it comes to the problem of modifying the blockchain. By computing the collision, it is possible to obtain a so-called *trapdoor*, which can be later used to modify a message in a transaction without changing its hash checksum at the same time. This means that the root of the Merkle Tree is not affected by changing one of the nodes.

Implementation is free of key exposure problems which could be very dangerous—on finding one of the collisions, it would be possible to determine other collisions or get a hold on secret keys, so-called trapdoors, which would enable modification of blocks for potential adversaries. The authors also show the possibilities of compressing the multiple blocks to a smaller number of blocks and guarantee *anti-persistence*—that is, no information about the present state of blockchain can be deduced from its past state. When it comes to managing the trapdoors, it is proposed that the central authority takes care of collision computations in private blockchains. In a permissioned blockchain, trapdoor keys can be distributed among blockchain participants, and collisions can be determined by using Multiparty Computation Protocol (MPC). Public blockchains can be treated similarly to permissioned ones, but secret keys can be distributed only to parties with significant hashing power rather than all users in the network. The reason behind this is that the most popular blockchain, Bitcoin, already assumes a majority of trusted people. It is, however, that trusting a few parties can be dangerous in some scenarios.

This approach is one of the less complicated solutions for blockchain mutability problems when it comes to changes in protocol. It has many strong perks, inter alia, its compatible with most of the currently used blockchains—authors showed the possibility of integration with Bitcoin cryptocurrency and their approach is undependable of the consensus algorithm. The way of passing and reconstructing the trapdoor keys is provided for all types of blockchain settings: private, permissioned, and public. In addition, it would be better if trapdoors could be stored without having too much authority to prevent the possibility of the keyholder abused power. Authentication of modification requester is proposed by providing a secret key, which can be troublesome when the key is stolen or lost. The consequences in that situation are unpredictable and out of control.

#### Chameleon hash with secret sharing

Ashritha et al.^28^ are taking advantage of chameleon-hash as well but instead of backward compatibility, they focus more on the granting modification power to more users and giving an insight into the new, post-redaction content in the block. This could be very helpful when changes are needed and the block publisher has left the network or wants to write illegal data in the chain. The system requires approval of the block publisher, but it can be lifted after some time or any other predetermined trigger like the leave of a keyholder from the network. After getting the ephemeral key from the publisher in one way or another, the validators can team up to reconstruct the second ephemeral key and redact the desired transactions in the given block. For divided the trapdoor key among users, Nonlinear Secret Sharing is used. Its design is able to prevent the Tompa-Woll attack, in which one of the validators submits false data, so only they are able to obtain the secret key, while the others are incapable because of the missing true data from the malicious validator. Similarly to the previous solution, for reconstructing the key, Multi-Party Computation is used. To make sure that the edited message sent during the process of editing the transaction or the whole block is not tampered with, authors take advantage of Digital Signature.

In a permissioned blockchain, it is proposed that the trapdoor key is shared between all permanent validators, therefore, disregarding the existence of an ephemeral key. In a public blockchain, the presence of the key is justified. Entities participating in the network have a lower level of trust between them and the validators might not be only the most significant participants of blockchain regarding their degree of participation in the network.

Despite the interesting idea regarding secret key distribution and the possibility of modification to others by exposing the ephemeral key, there are some drawbacks. Insight of requested modification is undesirable when modified data are sensitive. Data could be encrypted, but then this verification process is unnecessary and does not give any benefits to the process. There is no scenario for appending illegal content to the blockchain before modification. If that kind of content is being sent to the blockchain, then the publisher can object to deletion or alteration of content. Of course, the ephemeral key can be released after some time, but some data should be deleted as quickly as possible and no other convenient triggers are proposed. In the same way, as Ateniese et al.^26^ proving publisher’s identity is done by presenting a secret key (in this proposal - ephemeral key) but in contrast, the key can be released after some time, so modifying the block is still possible but unfortunately not by publisher. This means that the publisher loses control over their data after a specified time. In summary, releasing the block publisher’s ephemeral key should be further analyzed and tested against a handful of scenarios to determine better logic for handling specific situations.

#### Linkable digital multi-signature

Instead of dealing with hash functions, Cai et al.^29^ base their work on specific blockchain implementations, heavily relying on cryptography tools. It utilizes the Proof-of-Space consensus of SpaceMint cryptocurrency. Blocks consist of proof subblock, signature subblock, and transaction subblock, all of them being encrypted. For deletion operation, linkable digital multi-signature (LDMS) with one-time addresses is proposed. The transaction sender can request removal by either revealing their identity or transaction’s content. The first choice requires the generation of two traceable signatures, which are verified by the rest of the blockchain users. If the operation succeeds, the public key will be revealed and deletion will be possible. The second approach is done by disclosing transaction content by the sender and generating a Pedersen commitment scheme, which is one of three algorithms used in the Confidential Ring Transaction Protocol (Ring CT Protocol). The next members of the network check whether the given value is valid within the related block. As previously, success means that deletion can be performed. During the removal process itself, LDMS is being used, and transaction contents are replaced by signature. At the end, the new state of chain is broadcast to the whole network.

The approach is unique compared to previous ideas. It does not center around hashes as it is simply not utilizing them in the same way as previous ideas based on chameleon hashes because of the specific consensus protocol. Despite an interesting solution and elegant usage of the Ring Confidential Transaction Protocol and Pedersen commitment, there are some weak points. The first thing that comes to mind is the complexity of the solution. The specific structure of blockchain excludes its usage in already working networks and the creators of new ones might be discouraged from using this approach as it is hard to understand without proper cryptographic knowledge. Furthermore, every transaction in the block has to be done by the same sender, which might not be desirable for some implementations. Revealing some information might also be problematic in some scenarios, e.g. when data stored in transactions contain personal information, e.g. biometrics that can reveal the identity of the sender. In this situation both sender identity or transaction reveal means exposing sensitive data that shouldn’t be exposed. The big problem is the lack of proof of concept of the solution by the authors. The idea is complex. Implementing it on one’s own is extremely difficult compared to the other proposals.

#### Reconstructable ephemeral key with Proof of Redaction

In Sartori’s concept^30^, the main goal of the solution is to provide evidence of the alteration and have minimal impact on the block when modifying it. To achieve their objective, they use two ephemeral trapdoors based on chameleon hashes which allow mitigation of giving every entity the possibility of computing collisions after computing the first one for the given public key. By using a commitment scheme and secret sharing, one of the ephemeral keys can be reconstructed from the rest of the nodes in case the transaction sender refuses to delete, for example, illegal content, either by losing the key or leaving the network. By proposing Proof-of-Redaction, the solution gives the ability to track modifications in the chain and confirms made redactions. To combat malicious parties, Sartori proposes committing randomness into the proof-of-reaction to prevent additional redaction of the transaction just before the approval.

The approach presented is very interesting. The big asset for sure is the in-depth analysis of the problem and considering many options to solve it. The presented implementation in Golang is also a great plus. Unfortunately, the concept has some features which might be undesirable. For example, one of the questions is should one of the ephemeral keys be shared across the other nodes. Authors justify their decision by situations like key loss by the transaction sender or sender’s lack of engagement in the protocol. Many of their goals are based on GDPR, but it is difficult to see the situation when after losing the key, other participants would propose modification on the transaction sender’s behalf leaving the personal data in the blockchain without any way to delete it. Also, the second secret key seems to be the backup plan that will not be executed anyway because of the lack of the triggers for it. Lastly, the implementation in Golang could be shared in some form of a public repository to replicate the results. The code provided is incomplete for running the solution without much effort.

#### Erasure database

As opposed to the work described so far, Florian et al.^31^ focus on local erasure, omitting changes in the transaction protocol itself. The authors present the concept of *functionality preserving local erasure (FPLE)*. Any network participant can mark data for deletion or replacement. The new transaction contents are then stored in the erasure database, and the former local content can be deleted. This leads to a problem, where new transactions in the blockchain refer to the deleted ones. To defuse this situation, the authors enforce the rule, which says that transactions do not need re-verification. The old ones, dependable on the deleted ones, are valid, while the new ones, created after erasing referenced old content, are invalid.

The local approach to the problem seems out of place when it comes to modifying blockchain as a global effect when taking into account what all participants want. But in this case, the authors provided a good reason for their idea. When it comes to deletion requests, be it by law such as GDPR or simply prohibited content, the most important is **not what the content is, but who is storing and distributing it**. As correctly stated, there is no guarantee that everyone deletes or alters the requested content. Even if changes would be enforced globally, some entities could still make database snapshots, preserving old content. By this erasure procedure, one can dispose of problematic data while still being a rightful member of the network. This solution is even more interesting because it does not require any form of authentication or trusting other entities. The erasure database can also be a decentralized database in the form of a blockchain. Still, the solution is not checked against some possible scenarios, which can be a threat to the system, such as stealing the deletion possibility and spamming the database with unwanted content. Nevertheless, the authors provided a link to the public code repository, which is rare in this kind of work and always welcome.

#### Blockchain as authentication and authorization platform

One of the proposed solutions to the blockchain modification problem is a workaround from Truong et al.^32^ where blockchain, based on smart contracts, is actually an authentication and authorization platform. All data is stored on a separate entity named resource server, and access to it is regulated by the Access Control List (ACL) managed by users participating in the blockchain network. Owners of data are able to grant create, read, update, or delete access to third parties by executing one of the proposed smart contracts. After that, the specified third party receives a token that can be used to manage data stored on the resource server. The blockchain additionally stores pointers to protected data and their hash checksums. This means that the data under the protection of GDPR is not essentially stored in the blockchain itself. Therefore, the ability to redact the blockchain is unnecessary.

While this approach enables modification of data, it is not strictly related to the topic of modification of the blockchain itself. That is why it has been called a workaround. As data is stored somewhere else, the system is more vulnerable to cyberattacks such as a DDOS attack or a Ransomware attack. In case of failure to protect the data, data owners are left only with the checksums and pointers to non-existing information, or in a worse scenario, the data can be stolen without noticing it. Still, the big asset is the shared code by the authors that enables one to test a solution by oneself, and the blockchain’s role in the concept is crucial.

#### Tree structure

A linear chain of blocks is something that limits mutability to a meaningful degree. Kuperberg sees this and proposes a tree structure where every subtree coming from the root is related to a single business context^33^. That makes deletion of data much easier and less impactful on the way of how blockchain has to work (like securely storing ephemeral trapdoors in the chameleon-hash strategy). In the proposed approach, the author determines the scopes, which are effectively sets, related to every combination of entities, for example, organization A, organization B, person A, and person B make 16 sets (the relation is everyone with every subset from the remaining participants which can be written as $2^4$), including the empty set. In that way, the root of the tree takes the role of the genesis block and allows linking to it up to 16 subchains depending on the business operations like transferring currency to organization A by person A, transferring between organizations A and B, persons A and B, etc.

Kuperberg’s idea is quite easy to comprehend and to work with if one omits problems like how to handle data integrity, authorized voting, etc. that are not strictly related to the blockchain itself. However, there are some drawbacks that have to be pointed out. Dividing one long chain into many smaller ones makes them significantly shorter, especially when a given business context rarely occurs. That gives an opportunity to forge the chain by malicious users with large mining power. This approach also does not consider blockchain with the external world, e.g. the medical industry. For example, if a patient would gain discounts on some drugs based on the history of the disease (which is considered to be verified and true as it is stored on the blockchain), deleting the whole data is equal to losing benefits in the real world. The deletion request can be issued because of factors like privacy concerns, personal data change (modification would have to be done by deletion and adding new data), or even things independent of the patient, like data leak or company bankruptcy. Even if we consider things like this acceptable, some data are necessary for one party, e.g., the hospital would need information about what kind of resources has it used on the patient that is no longer in the system. Anonymizing the data by deleting only the person’s related information would keep these insights intact. The last thing worth mentioning is the growing blockchain structure. The number of subchains is $2^n$ where $n$ is the number of participants. Managing this kind of structure is very troublesome, as it can grow in breadth very rapidly. This implies that the transactions will be scattered, so the chains connected to the genesis block will be short and easy to forge if not properly secured. Even though there are some problems, many of them can be dealt with by using references to some data to enable collecting nonpersonal data or using the system in a more restricted environment as a permissioned or private blockchain.

### GDPR compliant solution based on Reference-based Tree Structure

This section is dedicated to proof-of-concept based on extended Kuperberg tree structure blockchain^33^, as the author stated any sort of implementation is missing at this moment and the proposed solution seems promising. Along with the proof-of-concept, there are some proposed modifications in the form of *Reference-based Tree Structure* (RBTS) which could benefit this solution, as described later.

#### Tree structure design

The tree structure is very similar to the one presented by Kuperberg^33^. People and organizations taking part in the blockchain network are grouped into sets of context scopes which are effectively subsets of the set of all participants, including an empty set.

**Figure 1:**
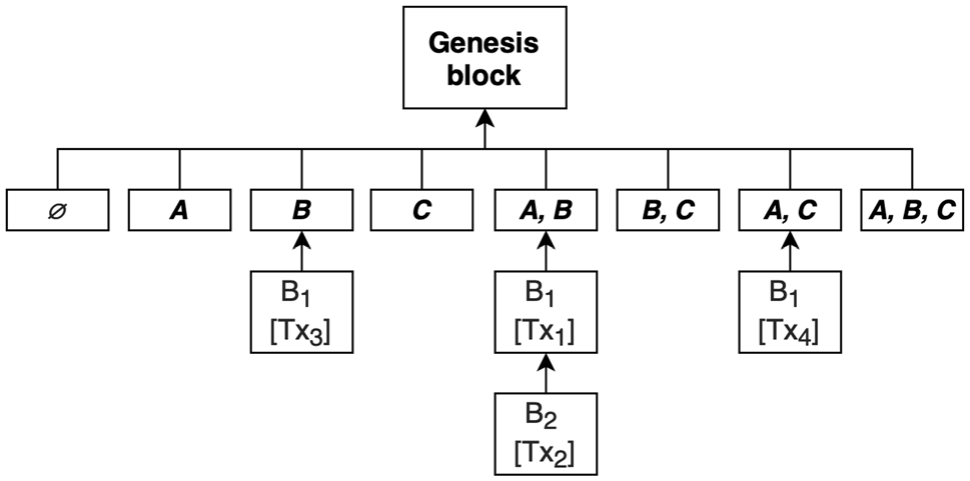
Blockchain has multiple subchains derived from the participants of the network for every business scope. Here is presented an example of blockchain with participants *A*, *B* and *C*.

Every context scope has a dedicated subtree (also called a subchain) with a root of the genesis block. This kind of approach brings a total of $2^n$ sets, where *n* is the sum of the number of persons and organizations. An example is provided in Fig. 1.

Of course, this means that with the growing number of participants, the number of subchains grows very rapidly. This should be a problem as for the most part business operations consist of a small number of participants, and some individuals can be represented by one, same organization. This means that, in reality, the possibility of $2^n$ in the blockchain is almost impossible unless only a few entities take part in it.

In this proof-of-concept, the subroots for the chains (the roots for the upcoming blocks for the given business context) are present in order to make it more clear on how it is working and to validate the correctness of the solution. Additionally, organizations and persons have been combined and represented as participants instead of distinguishing them as it does not matter in this case. This implementation is able to create an appropriate blockchain for as many participants as one puts in the config file, however, in the following example tests will be done on four participants for more clarity in the presented output.

This proof-of-concept utilizes the Proof of Work consensus algorithm as it is simple and most widely used in popular blockchain applications. Using other consensus algorithms is possible and should not cause much trouble. The blockchain requires an object of a Consensus class in its constructor, so one can make use of polymorphism and extend the abstract class by implementing the needed consensus and then passing it as an argument during the creation of the blockchain.

#### Computational complexity

The more blockchains grow into new blocks, the more computational complexity that impacts their efficiency. The complexity of computation, or simply, complexity, determines the resources needed to run a given algorithm. The main metrics for measuring them are memory and time. In this research, the focus is on time complexity without taking into consideration space complexity. The reason for this is the nature of operations. Appending blocks and deleting subchains have neglectable or non-existent space complexity by themselves (looking for element to might require some resources though) while the most expensive are permutations that do not take place in blockchain for it is an immutable data structure.

To classify the complexity of the further functions presented on the blockchain, the *big O notation* will be used, which can also be referred to as the *order of the function*. In computer science, the definition can be formed as follows.

Let *f* be a real or complex value function and *g* be a real value function. They both have to be functions of positive integers to nonnegative real numbers. The result of *g*(*x*) must be strictly positive for large enough values of *x*. Then, one can write^34^:

1 $$\begin{aligned} \exists _{M} \forall _{n \ge n_0} : f(n) \le Mg(n) \implies \lim _{x \rightarrow \infty } f(x) = O(g(x)) \end{aligned}$$

For particular cases finite ranges can be tacitly excluded from both *f*’s and *g*’s domain by choosing large enough $n_0$, for example *log*(*n*) is undefined at $n = 0$.

Speed of instructions on the blockchain should be high, as much more information will be appended than deleted which implies that input to functions will grow over time. It should be noted that changing the state of blockchain can be only done by deleting content with presented implementation - no alteration is possible. That’s why the computational complexity is crucial to mention when it comes to deleting the subchains as it might become a major bottleneck when not taken into consideration.

#### Deletion process

**Figure 2:**
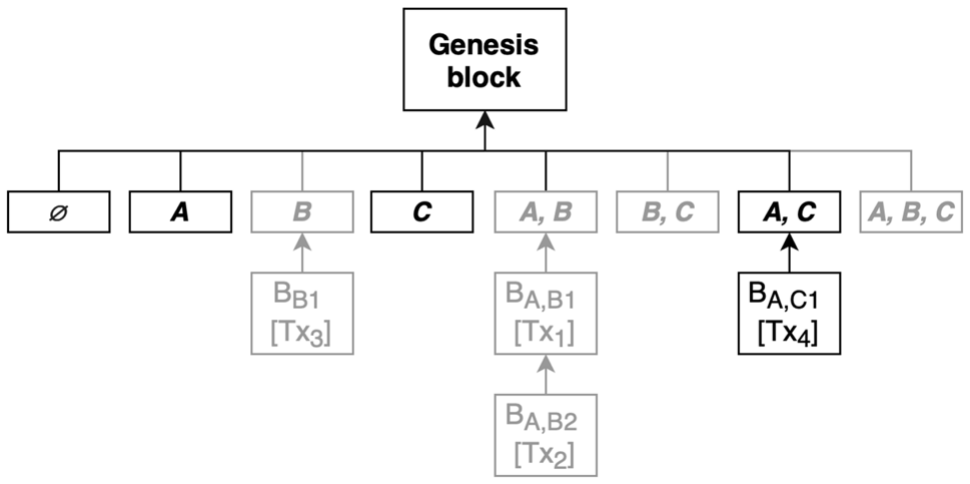
Leave of a participant implies deleting all of their shared subchains marked in this example with a gray color.

Every subtree (also referred to as subchain) in presented implementation is assigned to a key of a dictionary which is a tuple of participants related to it. These keys and bounded with them subchains are put in the dictionary which also a hash table. Deleting any subchain is computationally cheap, as the average complexity of this operation is *O*(1). Nonetheless, this approach requires deletion of every subchain related to the participant that wants to leave the network. If the list of all participants is available we can derive the keys of the dictionary with *O*(*n*) average complexity where *n* is the number of all participants, otherwise, it’s necessary to find all the related subchain keys in the set of all the keys ($O(2^n)$ complexity). Reference variation of the solution provides complexity of *O*(1) and will be described in next section. Example diagram showing the blockchain state after deletion can be seen in Fig. 2.

This kind of deletion doesn’t have any security logic in this implementation as there are many ways to wrap this process in a secure authorization mechanism that will be responsible to validate the deletion proposal by one of the engaged parties. Still, the solution presumes that every party that is sharing the sub chains of the leaving individual needs to agree on abandoning the network. The permit is not required unless there is at least one block mined on the shared chain and unless implementation of the blockchain delays the creation of shared chains till the time of mining the first block in them.

Other ways to delete the data, be it by removing the last block in the subchain, prefix or suffix truncation^33^ has not been implemented. Deletion of the last block is quite problematic, due to possibility of the ongoing preparation of the next block and bound to it transactions coming after it. Allowing this kind of data manipulation would require special consensus, as the mining block in the blockchain is considered final and can significantly slow down the blockchain protocol^33^. Any kind of truncation, like prefix or suffix one, is achievable, but requires changes to the protocol itself. Nevertheless, with the proposed structure, applying changes is much easier and less problematic due to not operating on one chain, but multiple ones assigned to given context scopes instead.

#### Reference variation

The main downsides of the presented solution, inter alia, are: high complexity of subtrees removal and losing some of the data that can be useful to some parties who made transactions with participants who just left the network. Information about the subjects of transactions or their metadata might still be useful even when they do not point at the exact individual, with whom they were agreed.

**Figure 3:**
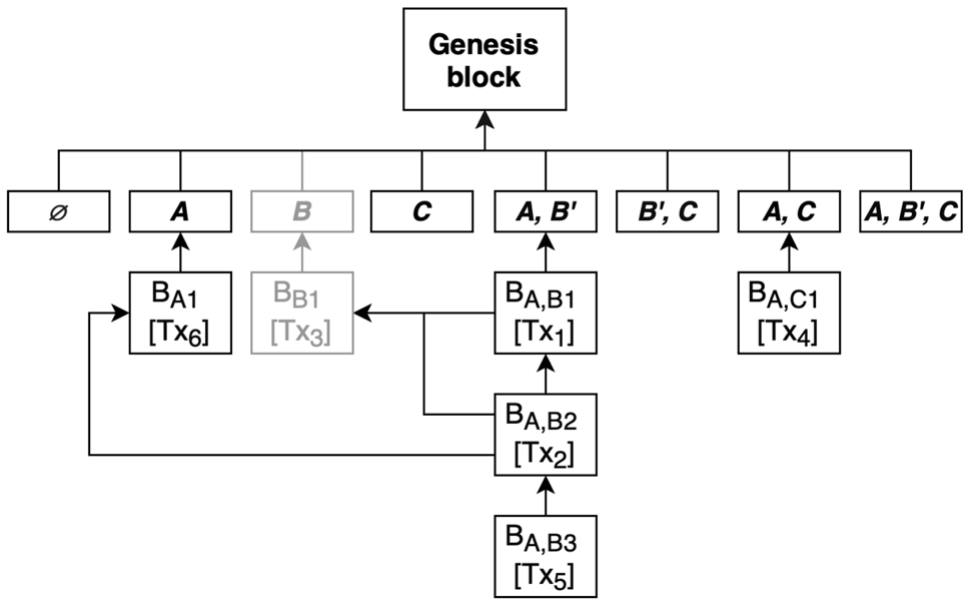
Instead of putting individual’s sensitive data in shared chains, it can be put in one party’s subchain and then pointed to from the other, shared transaction. Deleting only the subchain of the leaving party is sufficient.

To mitigate both drawbacks, a variation of the solution is proposed, which will be referred to as reference variation. This approach, for the cost of mining multiple blocks instead of one, allows one to leave the blockchain network without disrupting the structure of the shared subchains and deleting only one assigned strictly to the leaving party.

**Figure 4:**
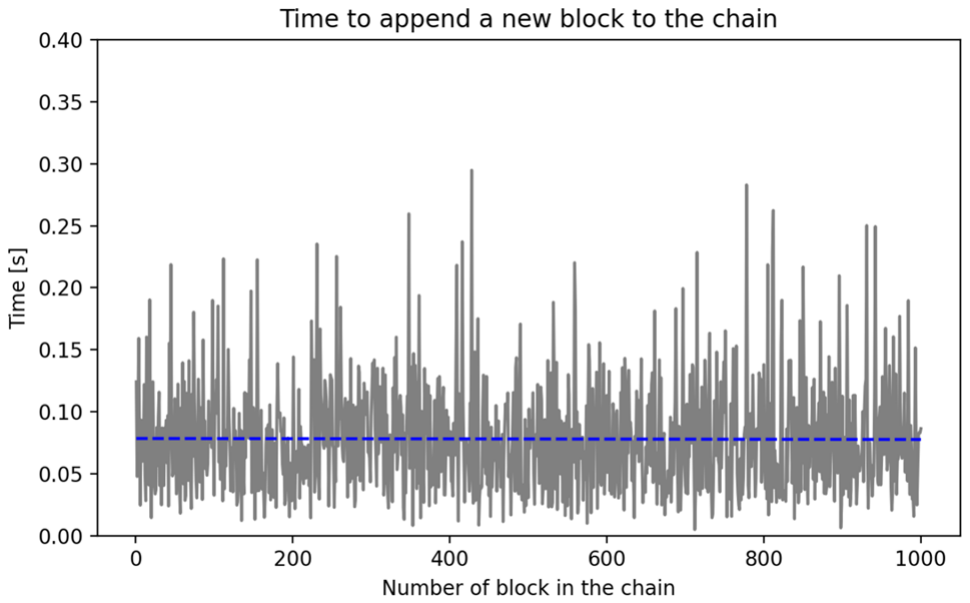
The length of the chain doesn’t impact the time of appending a new block.

This had been achieved by enabling to pass references to transactions and blocks when creating transactions. All crucial information about individuals has to be mined on their subchain first, so it can be used later when making transactions in a shared chain. When multiple parties make mutual transactions, they refer to the transactions in blocks, in their individual subchains. In that way, leaving the network does not require the approval of every party involved in the shared subchain. The data of leaving entity can be erased, but their identifier name in every shared chain is modified by appending the ’ character at the end in order to distinguish deleted users from those still in the network. Every party that had transactions with the former participant can still see how many transactions were made and what they were about unless those data were considered sensitive and put on the individual’s subchain. It is debatable if the empty shared subchains should be also deleted. On the one hand, empty subchains are metadata itself, and on the other hand, it might lead to an excessive tree structure. The problem does not exist when the creation of shared subchains is postponed until the mining of the first block. Nevertheless, this does not have an impact on the deletion itself and can be adjusted to one’s will according to the implementation of the blockchain. The example of the presented approach can be seen in Fig. 3.

#### Controlling the blockchain

The control of the blockchain could be done in one of the following ways. One of the approaches would be hardcoding the example into Python code. This approach had been dismissed as providing input in this way is considered a software antipattern. The program could also take interactive input from the user, but this avenue had also been declined. Typing commands to make changes in the program is simple for the end-user, but an interactive form can make operations in it much less reproducible. That is why the instructions are chosen to be written in the config.yaml configuration file following the YAML syntax.

**Figure 5:**
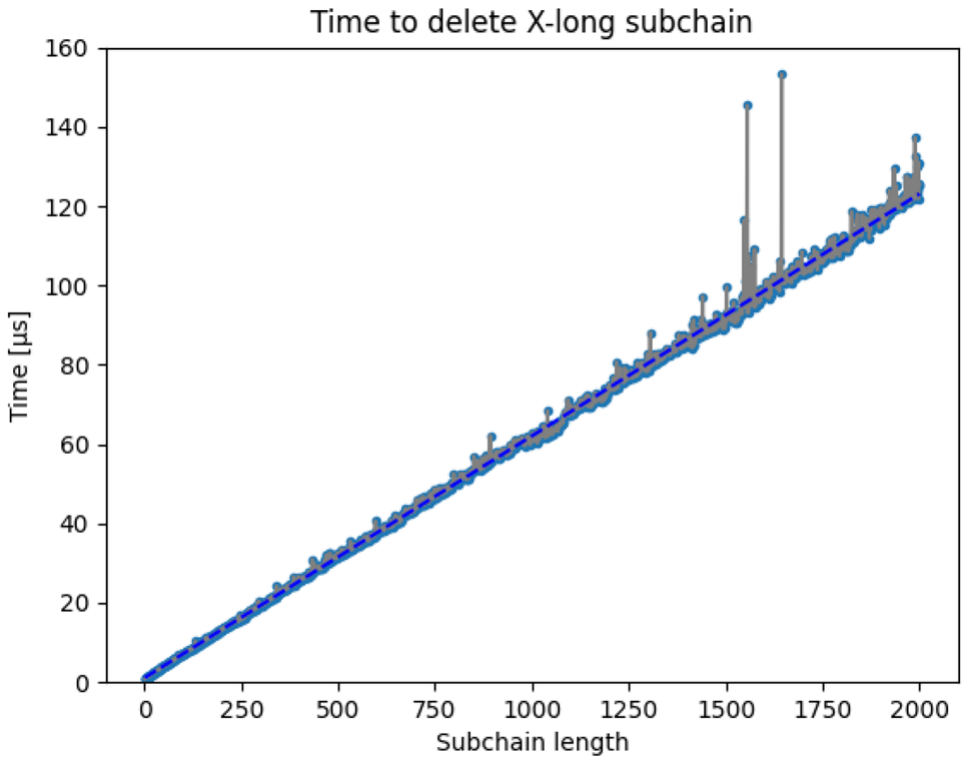
Time to delete subchain is directly proportional to its length.

This kind of parsing enables easy reproducibility for various configurations. The file can be shared with anyone without possible changes to the program logic and an additional explanation on how to reproduce the research. YAML format had been chosen because of its readability more than other popular formats such as XML or JSON. The only significant downside of this method of input is the lack of possibility of passing commands in the runtime. It is acceptable though as it is not so important in the proof-of-concept. The user is able to determine participants of the blockchain and flow of the program like printing the state of the blockchain, adding transactions, mining blocks, and of course, deleting the particular subchain.

## Experiment results

In order to confirm the solution’s ability to delete content from the blockchain in the previously described manner called reference variation, a scenario has been formed. First, the initial participants will be added to the system, *A*, *B*, and *C* followed by the mining of the genesis block. Next, a transaction with some information about the participant *B* will be put into the block, which will be extracted and placed on the subchain *B*’. Subsequently, another transaction will be formed as an agreement between *A* and *B* referencing the previously created transaction and block and put into a new block mined and placed on subchain *A*, *B*. Finally, the state of the blockchain will be shown, the participant *B* will leave the blockchain, and to verify the state, the blockchain will be presented again in the console. The experiment will be considered successful, when only the *B* subchain will be deleted, as opposed to Kuperberg’s^33^ solution where every subchain containing the entity that leaves the blockchain should also be deleted. Instead, the related chains to *B* should be renamed by changing the former participant’s identifier to $B'$.

Deleting personal data of *B*, respects GDPR’s *right to be forgotten*, therefore, making blockchain compliant with the regulation. In addition to verifying the output of the software, a performance test was performed. The complexity of adding a new block to one of the context chains is *O*(1), as expected. This can be seen in Fig. 4, in the form of a graph.

Unfortunately, it is not possible to achieve the same time for adding every block to the chain. The time spent on mining a block may vary due to taking a longer or shorter time to mine the block, hardware optimizing the power usage, the limited list capacity that needs to be extended, or any other external factors. Nevertheless, the blue line shows the trend that length of chain does not impact time to append new blocks to it. Measurements were also made for a situation where the blockchain participant leaves the network. The deletion of one of the subtrees is implemented as the removal of an entry from the Python dictionary. This operation has a complexity of *O*(1).

The reason for this might be Python’s implementation for removing the value under the key in the dictionary. In this case, a list has to be removed, so it is important to keep in mind the time needed for memory management of the program. Still, the results of the benchmark are satisfying as deleting a chain does not take longer than one second even when it comes to the long chains with block number reaching 100000.

**Figure 6:**
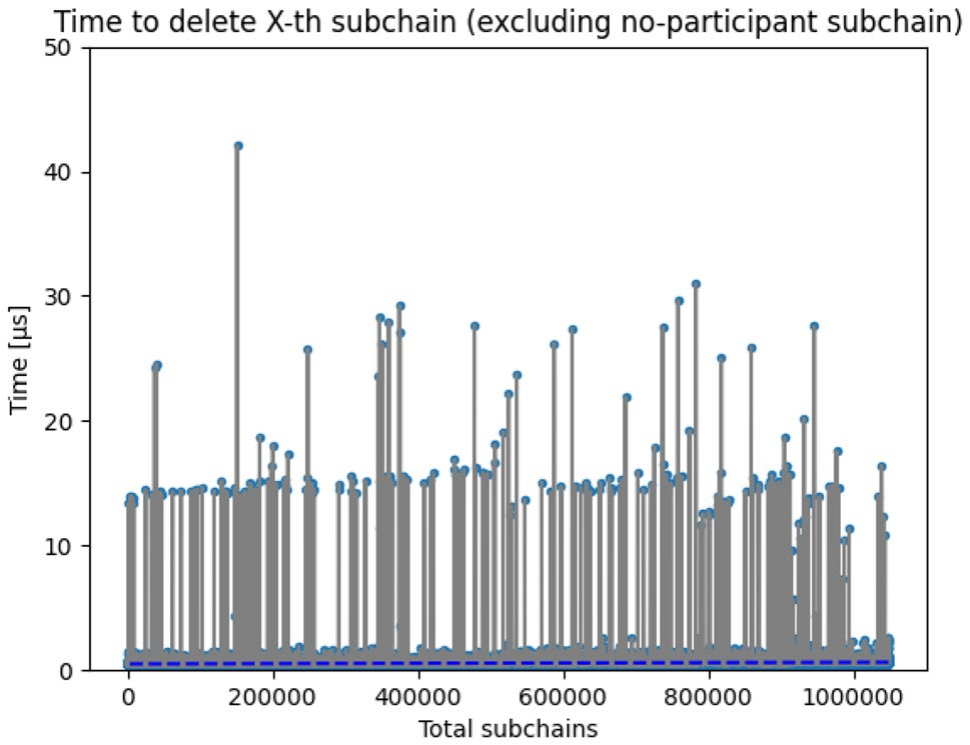
Time to delete subchain is not related to the number of other subchains in blockchain.

It is worth noting that the time to delete any of the context chains is rapid. In the tests carried out, it can be seen that although the time to delete the subchain is linear with respect to the length of the subchain, it is still small, as seen in Fig. 5. Time to delete a subchain does not exceed 160 microseconds for lengths up to 2000 blocks. There are 2 visible spikes in the chart between lengths 1500–1750. This can be a result of using Python, which uses a garbage collector to deallocating memory. Inspecting context chains with the highest deletion times has shown no correlation between the time to delete and the number of participants in the chain.

The tree-structure of blockchain may result in a large amount of context chains. The proposed implementation considers this and makes time to delete any of the context chains not bound to total number of subchains in blockchain. Figure 6 shows the result of tests proving that the time to delete chain does not exceed 50 microseconds when there are 1 million of them present in the system.

In contrast to the other related works, this solution comes with a working implementation. In addition to that, the vast majority of solutions previously analyzed require interference from other parties to remove personal data from one party. Here, with the reference variation, the problem is solved as this approach does not require any approval of any other party, though it can be added if needed. Additionally, metadata can be preserved based on what is not treated as sensitive data, and therefore these data can be stored on shared chains. Lastly, this implementation does not impact performance for write operations to the blockchain, and deleting particular chains on demand is quick.

## Conclusion and future work

The aim of this paper was to analyze existing methods for enabling compliance between blockchain and the General Data Protection Regulation introduced by the European Parliament, then propose and implement proof-of-concept for one of the covered methods. The goal of the research has been achieved and the scope is fully covered. To achieve GDPR compliance, it is clear that some form of mutability in the blockchain is necessary to be able to fulfill potential deletion requests using the individual’s *right to be forgotten*. Many researchers are making their effort to find an efficient solution for this since the enforcement of the regulation, and many of them have distinguishable ideas—from simple ones like erasure database to more complicated ones like linkable digital multisignature. All ideas are discussed by pointing to their strengths and weaknesses. The common requirement in the presented concepts is some form of access control in order to steer the logic for deletion or alteration requests, which shows potential for smart contracts.

The key advantages of the proposed solution is a unique design that allows deleting data from the blockchain ’silently’ without interaction from other blockchain nodes and no impact on the performance of writing data into the database. Reproducibility is achieved by uploading source code, scripts data set and all raw data to the public git repository^35^ and containerized application with the use of Docker. The exhibited *reference variation* of the solution (RBTS), enables to point to the sensitive data on an individual’s subchain instead of storing it in a shared subchain, therefore, giving the data owner only ownership. That means deleting one’s content can be done anytime by the personal data owner. Performance tests show no impact on the time to append new blocks in subchains, and the deletion operation itself is rapid, even when the chain is very long.

The solution has some downsides, though, like the use of a very simple blockchain without interfaces for distributed testing, or the only consensus in form of a Proof of Work algorithm. The testing scale is also troublesome as it is hard to determine how blockchain will act upon interaction with many users. The logic layer for the deleting process is also unspecified. However, the majority of problems can be resolved in the future. To solve them and make the solution more portable, it is proposed to implement the system in the form of smart contracts that can handle most of the mentioned issues. Additionally, changing the consensus algorithm is desired, like the Proof-of-Stake one, in order to reduce electrical energy consumption.

## Data Availability

Data sets (raw data, scirpts, source code) generated and analyzed during the current study are available in the github repository, https://github.com/matgd/tree-structure-blockchain/ and also are available from the corresponding author on reasonable request.
